# Interaction between nasal epithelial cells and Tregs in allergic rhinitis responses to allergen via CCL1/CCR8

**DOI:** 10.3389/fimmu.2025.1526081

**Published:** 2025-02-20

**Authors:** Jichao Sha, Maolin Yang, Yashu Lei, Liwei Sun, Cuida Meng, Dongdong Zhu

**Affiliations:** Department of Otorhinolaryngology Head and Neck Surgery, China-Japan Union Hospital of Jilin University, Changchun, China

**Keywords:** allergic rhinitis, nasal epithelial cells, regulatory T cells, cytokines, CC chemokine ligand 1

## Abstract

**Background:**

The airway epithelial barrier is the first defence against aeroallergens. Nasal epithelial cells (NECs) are vital in regulating innate and adaptive mucosal immunity in allergic rhinitis (AR). Tregs produce cytokines essential for the immunomodulatory activities in allergen immunotherapy. Understanding the relationship between NECs and Tregs in the airway hyperresponsiveness network is essential for developing novel treatments.

**Methods:**

Using an *in vitro* human Treg-NEC co-culture system of AR and health control group, the chemokine expression profiles of NECs were examined using immunohistochemistry, RT-PCR, and ELISA, and functional surface markers of Tregs were detected using flow cytometric analysis. Correlation analysis was performed between cytokines derived from NECs and surface markers of CD4+CD8+Foxp3+ Tregs in the AR group after co-culture, including TSLP/CTLA4, CCL1/CTLA4, TSLP/CTLA4, TSLP/CCR8, and CCL1/CCR8.

**Results:**

CCR8 and CTLA-4 expressions after co-culturing were higher than single culture. Following Derp1 stimulation, TSLP, IL-25 and TGF-β expressions in the AR + Derp1 group were increased. CCL1 mRNA was lower in the AR + Derp1 group than control group. In the AR + Derp1 group, TSLP was higher, and CCL1 protein levels were decreased. There were no significant differences in IL-25, TGF-β and IL-10. When Treg co-culture group added, changes were similar to that observed in pNECs. After co-culture, CCL1/CCR8 was positively correlated in AR.

**Conclusion:**

Human pNECs can communicate with Tregs directly, CCL1/CCR8 may be the pathway between NECs and Tregs *in vitro* and may play a key role in the immune network of AR.

## Introduction

Allergic rhinitis (AR), characterised by upper airway hyperresponsiveness, is a major allergic disease of global concern. Its incidence has increased dramatically in recent years and is undertreated, even according to guidelines (1, 2). An aberrant type 2 response to aeroallergens may be owing to upregulated pro-allergic pathways and disrupted epithelial barriers. Exposure to allergens could lead to the release of epithelial-derived cytokines, such as thymic stromal lymphopoietin (TSLP), transforming growth factor (TGF)-β, and interleukin (IL)-25 (3). Nasal epithelial cells (NECs), as the first mucosal defence against inhaled allergens, are being increasingly studied but remain undefined. Allergen immunotherapy (AIT) remains the only disease-modifying treatment for AR, which induces immunological tolerance characterised by increased Foxp3+ Treg cell count and decreased sensitivity to Treg apoptosis (4, 5). Most studies have examined the effects of NECs on dendritic and T helper cells in the nasal mucosa. However, the relationship between NECs and Tregs in the network of airway hyperresponsiveness remain unexplored.

AR is triggered by the disruption of the nasal epithelium through the production of epithelium-derived cytokines such as TSLP and IL-25 (6). CCL1/I-309 is a member of the CCL chemokine family and is produced by several cellular sources, including airway epithelial cells (7). Previous studies have suggested that CCL1 may play a role in lymphocyte recruitment in allergic disease (8). CCR8 is an essential receptor for CCL1, and intrathecal injection of recombinant CCL1 into naïve mice induces hypersensitivity (9, 10). Notably, Tregs exhibit suppressive activity through CCR8 expression in mice and humans (11).

This study investigated the immunological features of human primary NECs (pNECS) and Foxp3+ Tregs to identify key molecules involved in their interactions. We focused on TSLP, TGF-β, IL-25, and CCL1 derived from NECs and CCR8 and CTLA-4 expressed by Tregs.

## Materials and methods

### Patients

The study cohort consisted of 40 patients with dust mite-induced AR (17 females and 23 males, aged 19–49 years, mean age 32.1 ± 6.3 years) and 40 healthy volunteers (15 females and 25 males, aged 26–32 years, mean age 28.9 ± 1.7 years). A clinical interview was conducted before the collection of peripheral blood and NECs. All participants were non-smokers, non-obese, without a diagnosis of an immune disease, and no history of recent infection. This study was approved by the ethics committee of our institute, and informed consent was obtained from all volunteers.

### Primary nasal epithelial cells

The pNECs were obtained from bilateral inferior turbinate under nasal endoscope by brushing (radius*length: 0.49 mm*1.2 cm, Pestro Healthcare Inc.). NECs were collected from each side, and the brush was gently turned twice and placed in an Eppendorf tube containing 1 ml phosphate-buffered saline (PBS) buffer. Cell suspensions were diluted to 1 × 10^5^/ml with 10% foetal bovine serum-BMGM medium (Lonza, Switzerland) and seeded into 35 mm cell culture dishes (Corning, USA). Once the monolayer of pNECs was confluent, Derp1 (5 ug/ml) recombinant protein or PBS was added to the groups. The adherent cells and cell suspension were collected after 24 h of incubation (37°C, 5% CO_2_) for subsequent co-culture and detection.

Subsequently, 40 patients with AR (21 males and 19 females, aged 27–45 years, mean age 31.7 ± 5.40 years) with positive house dust mite allergens and 30 healthy volunteers (19 males and 11 females, aged 20–52 years, mean age 30.3 ± 2.50 years) were enrolled. Subsequently, tests and analysis were used to detect: 1) the AR group, with Derp1 (ab73855, abcam), group A; 2) the AR group, with PBS, group B; 3) the healthy control (HC) group, with Derp1, group C; and 4) the HC group, with PBS, group D.

### Isolation and culture of human Foxp3+ Tregs with pNECs

Foxp3+ Tregs were purified from peripheral blood mononuclear cells through magnetic cell selection using a CD4+CD25+Foxp3+ Treg Isolation Kit (Miltenyi Biotec, Germany). The purity was approximately 75–80%, identified using flow cytometry with Foxp3-APC (eBioscience, USA). Subsequently, Tregs (3 × 10^4^ cells/dish) were cultured with confluent pNECs from a single donor.

### Co-culture of pNECs and Foxp3+Tregs

The pNECs-Foxp3+ Tregs co-culture model was established. Closer inspection and analysis were applied to detect these healthy volunteers: 1) NECs-Tregs co-culture group, pNECs were adherent, group A; 2) NECs-Tregs co-culture group, pNECs were suspension, group B; 3) Treg cells cultured in co-culture medium alone, group C; and 4) RPMI-1640 was used as blank control group of Tregs cultured alone, group D.

### Immunostaining

Foxp3 is a transcription factor involved in Tregs. Immunostaining of Foxp3 in the nasal mucosa (n = 10, 5 in each AR and HC group) was performed. The primary antibody was a rabbit anti-human Foxp3 polyclonal antibody (Wuhan Huamei Biotech Co., LTD, China). All sections were observed and counted under a microscope. Positive staining for Foxp3 was considered tan. Ten high-power fields (400×) were randomly selected from each section, and the percentage of positive cells in each high-power field was recorded. The average value was used to calculate the expression rate of the positive cells.

### Flow cytometry

Flow cytometry was performed on freshly isolated or stimulated Foxp3+ Tregs using the following antibodies: CD4-FITC (eBioscience, USA), CD25-PerCP-cyanine 2.5 (eBioscience, USA), Foxp3-APC (eBioscience, USA), CTLA-4-PE (eBioscience, USA), CCR8-PE (BioLegend, USA), and PerFix-nc Kit (no centrifuge assay kit) (Beckman Co. USA). The cells were analysed using flow cytometry (Accuri C6; BD Biosciences, USA), and 10,000 events in live cell gates were acquired in array analysis. The application settings were applied in each experiment to standardise the flow cytometric readouts over time.

### Quantitative real-time PCR

Quantitative real-time PCR was performed on confluent and co-cultured pNECs for TSLP (F 5’-GGCTGCCTTAGCTATCTGG -3’), IL-10 (F 5’-GCATTCTTCACCTGCTCCAC -3’), TGF-β (F 5’-GAGCCTGAGGCCGACTACTA-3’), IL-25 (F 5’-GTAGGGCCAGTGAAGATGGA-3’), CCL1 (F 5’-TTCACCAGGCTCATCAAAGCT-3’) mRNA, and beta-actin (F 5’-AAGAGCTACGAGCTGCCTGA-3’) in AR and HC groups (QIAGEN, German). Data were analysed using a real-time PCR (ABI7300, Applied Biosystems, USA) and ABI Prism 7500 SDS Software.

### Enzyme-linked immunosorbent assay

An enzyme-linked immunosorbent assay (ELISA) was conducted to determine IL-10, TGF-beta, IL-25, and CCL1 expression after Derp1, Tregs, or both, stimulated cell suspension in AR and HC groups using an ELISA kit (eBioscience, USA). The optical density was measured, and the detected protein concentration was calculated according to the standard curve.

### Statistical analyses

Statistical analyses were performed using the chi-square, Mann–Whitney U, or T-test. Statistical significance was set at P <0.05.

## Results

### Immunohistochemical staining of Foxp3 in nasal mucosa

**Figure 1:**
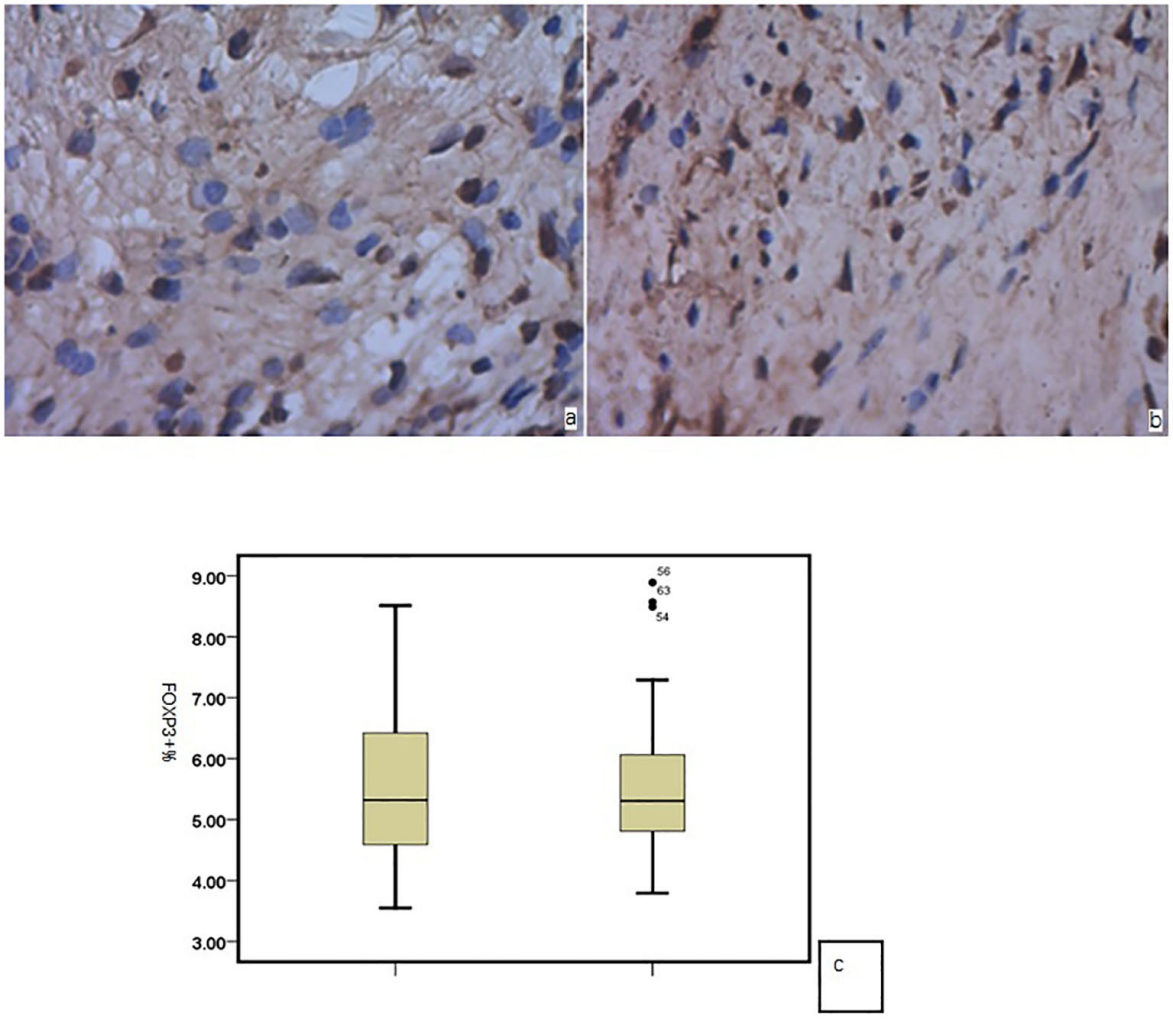
Foxp3 in the nasal mucosa of **(a)** AR and **(b)** HC; **(c)** no significant difference between the two groups.

In the nasal mucosa of AR and HC inferior turbinates, Foxp3 showed positive expression in the cell nucleus (brown) after immunohistochemical staining, mainly in the nucleated round mononuclear cells. There was no significant difference in the number of Foxp3-positive cells between the AR and HC group (P = 0.256) (**Figure 1**).

### Cell population and pNECs-Foxp3+ Tregs co-culture model

PNECs were obtained from 40 patients; the mean cell count is (2.84 ± 0.67) × 10^5^ cells per patient before culture. The 80% confluence rate was 40% (n = 40,16/40), and the average time from culture to confluence was 15.2 ± 1.25 days.

**Figure 2:**
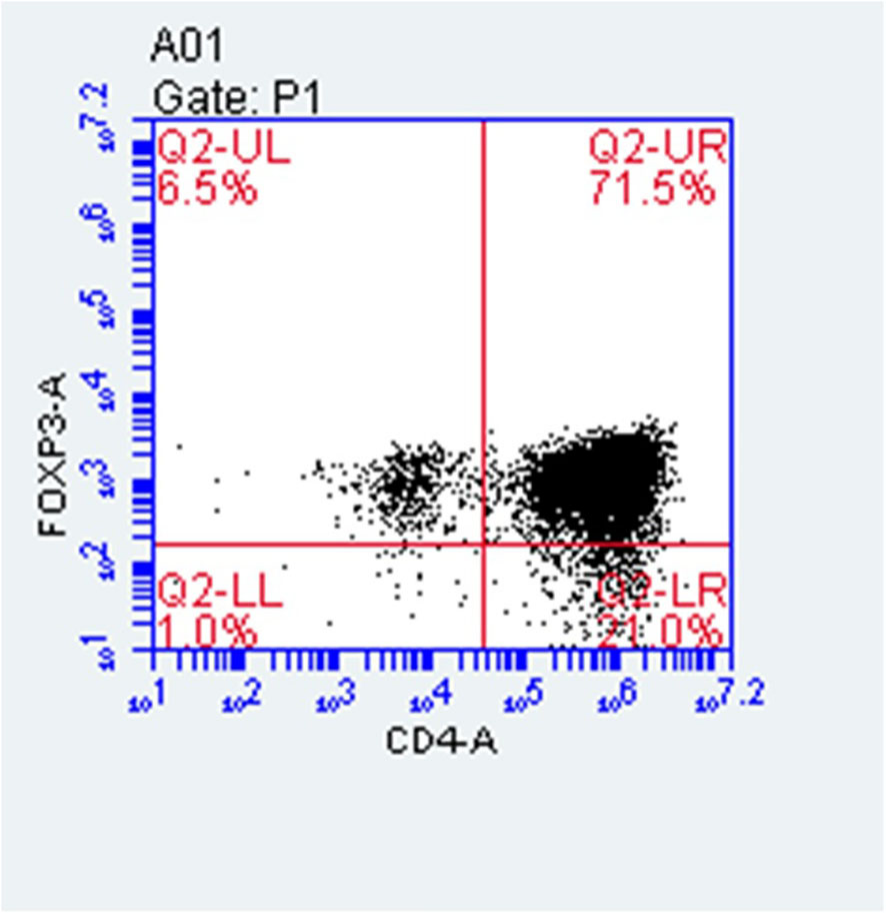
Purity of CD4+CD25+ Tregs isolated using immunomagnetic beads.

The average number of Tregs isolated by immunomagnetic beads in 36 patients was (1.35 ± 0.54) × 10^5^ cells per sample. The co-culture experiment required at least 4 × 3 × 10^4^ and 1.0 × 10^4^ cells. Therefore, the number of Treg cells after isolation was at least 1.3 × 10^5^. Only 16 of 36 patients met the requirement (44.4%, n = 36, 16/36). The purity of CD4+CD25+ Tregs isolated using immunomagnetic beads was 75–80%, as identified by flow cytometry using Foxp3 as a surface marker (**Figure 2**).

**Figure 3:**
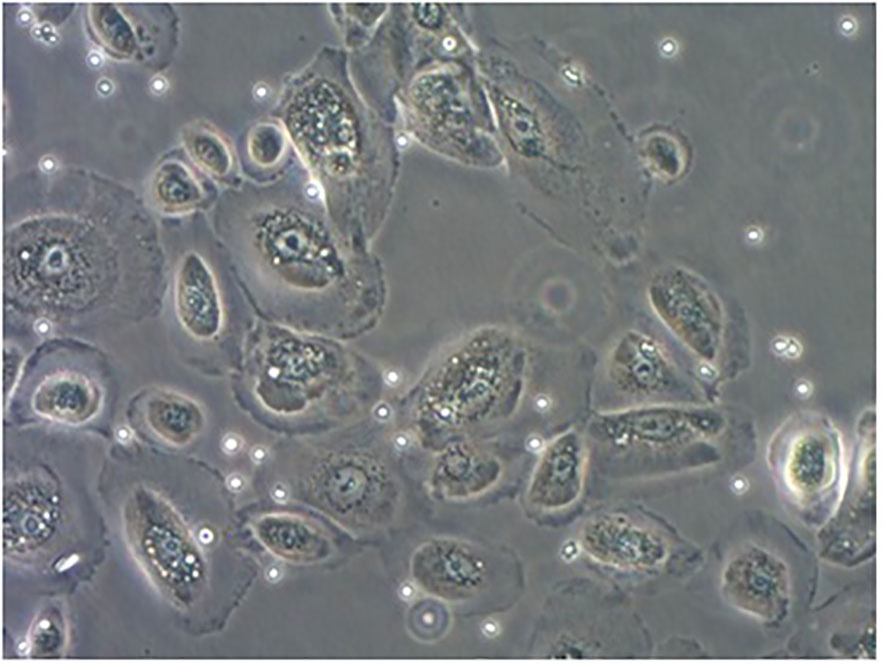
PNECs-Foxp3+ Tregs co-culture model. The adherent cells were nasal mucosa epithelium and the suspension was Foxp3+ Tregs.

The pNECs-Foxp3+ Tregs co-culture model was successfully established (**Figure 3**). In the last part of the study, the number of samples with 80% successful confluent NECs was 18 (n = 40) and 19 (n = 40) cases in AR and HC groups, respectively. After co-culture with Tregs from blood collection, co-culture models were successfully established for 16 patients in each group.

### Foxp3, CCR8, and CTLA-4 expressions of CD4+CD25+Foxp3+ Tregs cells in co-culture and single culture using flow cytometry

**Figure 4:**
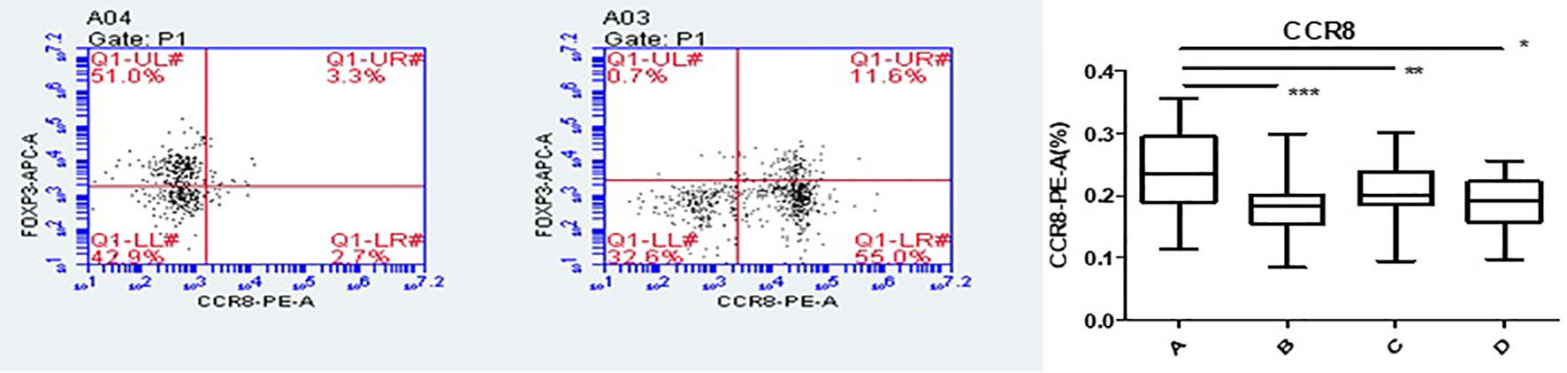
CCR8 expression of Tregs in group A increased following co-culture. *, **, ***p < 0.05 was considered statistically significant.

**Figure 5:**
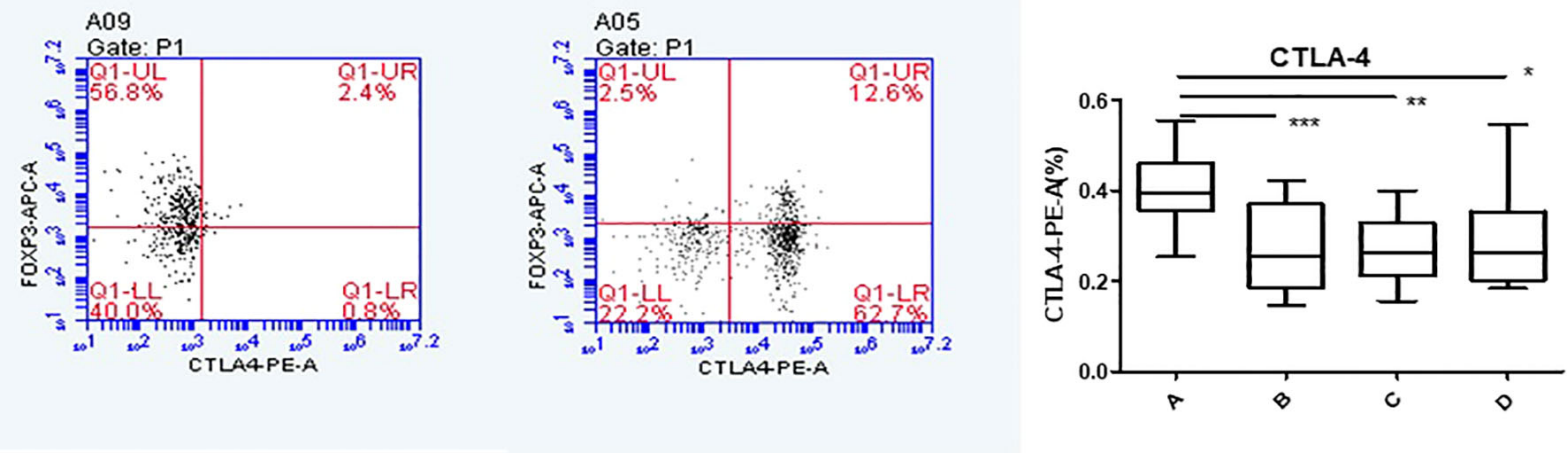
CTLA-4 expression of Tregs in group A increased following co-culture. *, **, ***p < 0.05 was considered statistically significant.

**Figure 6:**
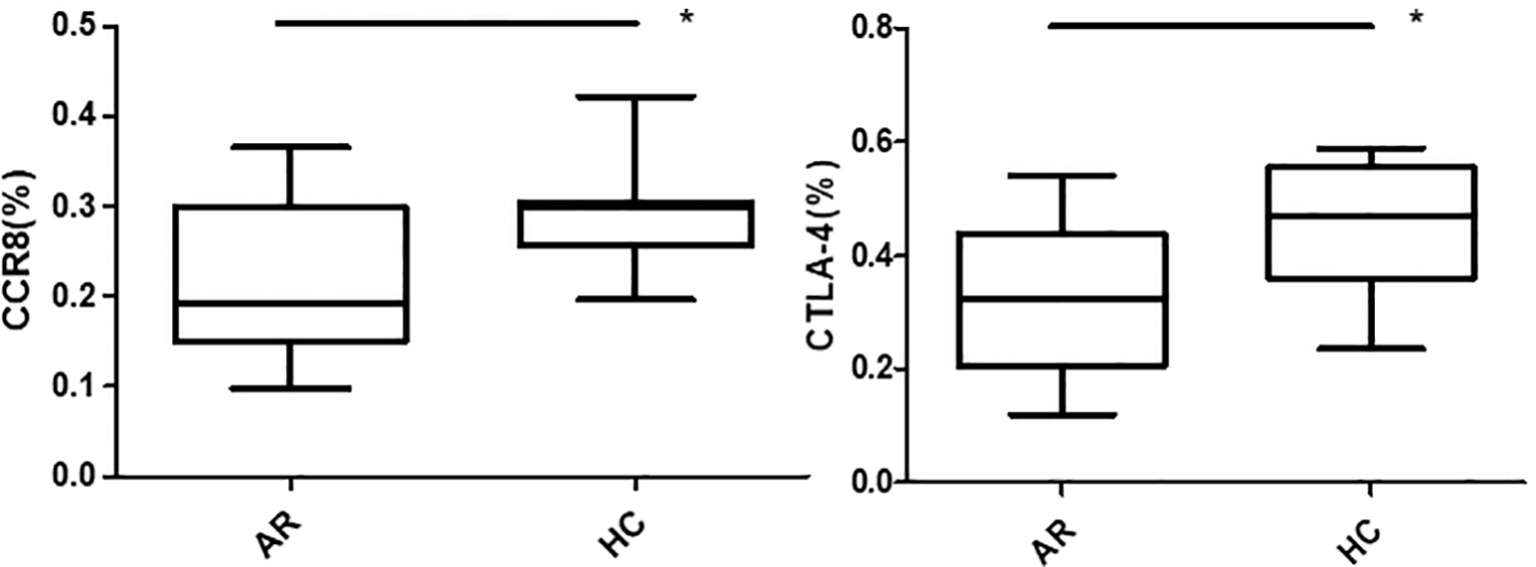
CCR8 and CTLA-4 expressions of CD4+CD25+Foxp3+ Tregs were lower in the allergic rhinitis group than in the healthy controls (P = 0.011 and P = 0.007, respectively). *P <0.05 was considered statistically significant.

Flow cytometry was used to detect HCs in all four groups. The Foxp3, CCR8, and CTLA-4 expression levels were compared between groups. There was no significant difference in the Foxp3 expression between groups A, B, C, and D (P >0.05). CCR8 expression was significantly higher in group A than in groups B, C and D (P <0.05). However, there were no significant differences among groups B, C, and D (P >0.05). CTLA-4 expression was significantly higher in group A than in groups B, C and D (P <0.05). There was no significant difference in CTLA-4 expression among groups B, C, and D (P >0.05) (**Tables 1**, **2**, **Figures 4**, **5**). When adding the disease group after co-culture, CCR8 and CTLA-4 expressions were lower in the AR group than in the HC group (P = 0.011 and P = 0.007, respectively) (**Figure 6**).

**Table 1:** Foxp3, CCR8, and CTLA4 expressions in four groups (x ± s).

	A	B	C	D
Foxp3, %	61.93 ± 13.16	65.80 ± 14.26	62.99 ± 13.53	62.57 ± 9.36
CCR8, %	39.94 ± 7.91	28.78 ± 9.91	30.48 ± 7.99	30.48 ± 7.99
CTLA-4, %	23.47 ± 6.71	18.34 ± 6.22	19.09 ± 4.23	18.91 ± 4.44

**Table 2:** P values of Foxp3, CCR8 and CTLA4 among groups.

	A, B	A, C	A, D	D, B	D, C
Foxp3	0.432	0.824	0.875	0.455	0.920
CCR8	0.001*	0.002*	0.002*	0.786	0.240
CTLA-4	0.033*	0.035*	0.031*	0.768	0.279

*P <0.05 was considered statistically significant.

### TSLP, IL-10, TGF-β, IL-25, and CCL1mRNA in pNECs

**Figure 7:**
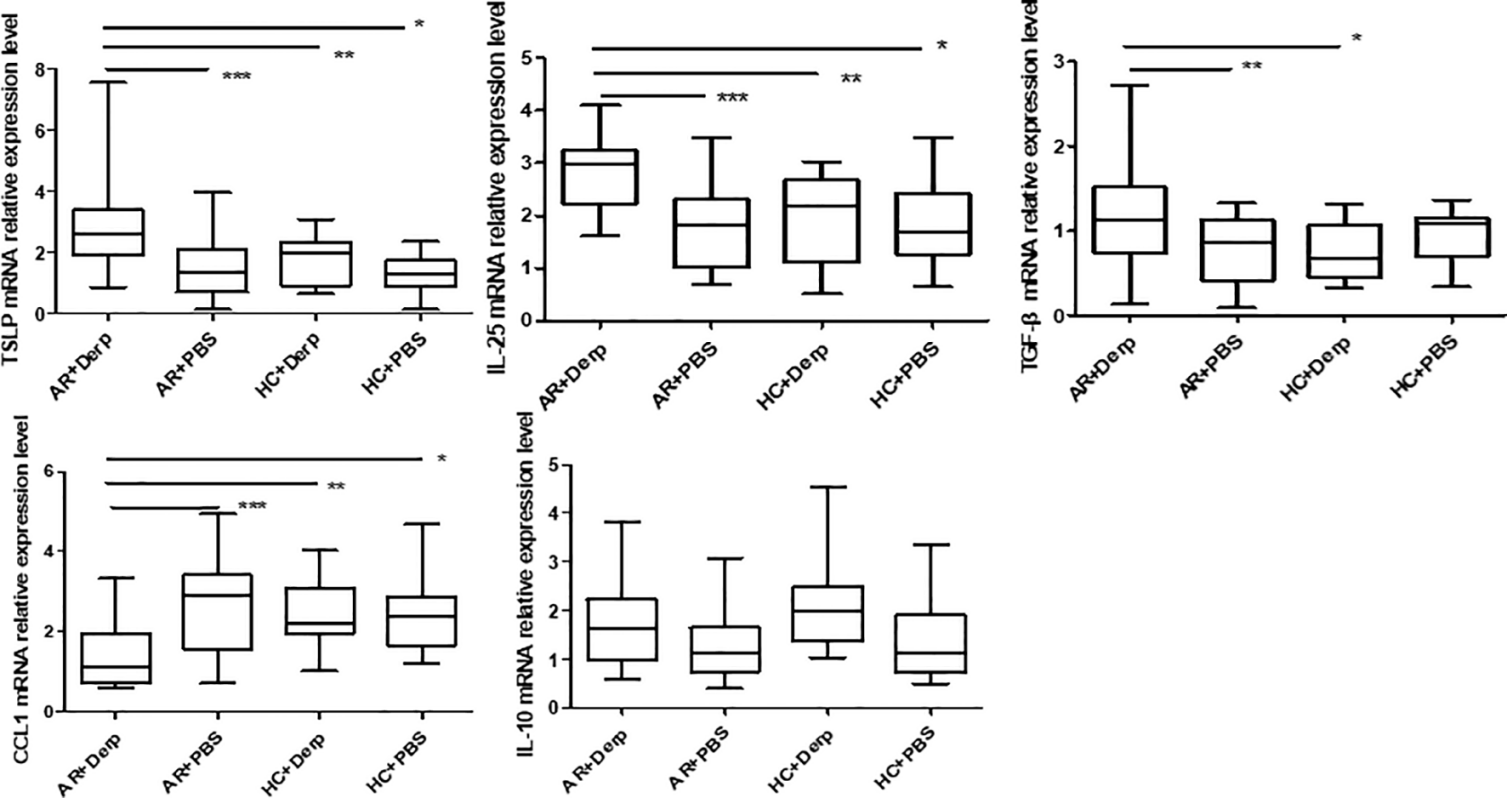
TSLP, IL-25, and TGF-β mRNA in pNECs in the AR + Derp1 group increased after Derp1 stimulation. CCL1 mRNA expression was lower in the AR + Derp1 group than in control groups. No significant difference was found in IL-10 mRNA. *P <0.05 was considered statistically significant.

After 24 h of Derp1 stimulation in pNECs, quantitative real-time PCR showed that the expressions of TSLP mRNA (P = 0.001, P = 0.01, and P = 0.000), IL-25 mRNA (P = 0.000, P = 0.006, and P = 0.001), and TGF-β mRNA (P = 0.016, P = 0.023, and P = 0.250) in AR + Derp1 group were increased. CCL1 mRNA was significantly lower in the AR + Derp1 group than in the control groups (P = 0.001, P = 0.023, and P = 0.08). There was no significant difference in IL-10 mRNA expression between the two groups (P = 0.141, P = 0.265, P = 0.358) (**Figure 7**).

TSLP, IL-25, and TGF-β mRNA in pNECs in the AR + Derp1 group increased after Derp1 stimulation. CCL1 mRNA expression was lower in the AR + Derp1 group than in control groups. No significant difference was found in IL-10 mRNA.

### TSLP, CCL1, IL-25, IL-10 and TGF-β in cell suspension of pNECs and co-culture by ELISA

**Figure 8:**
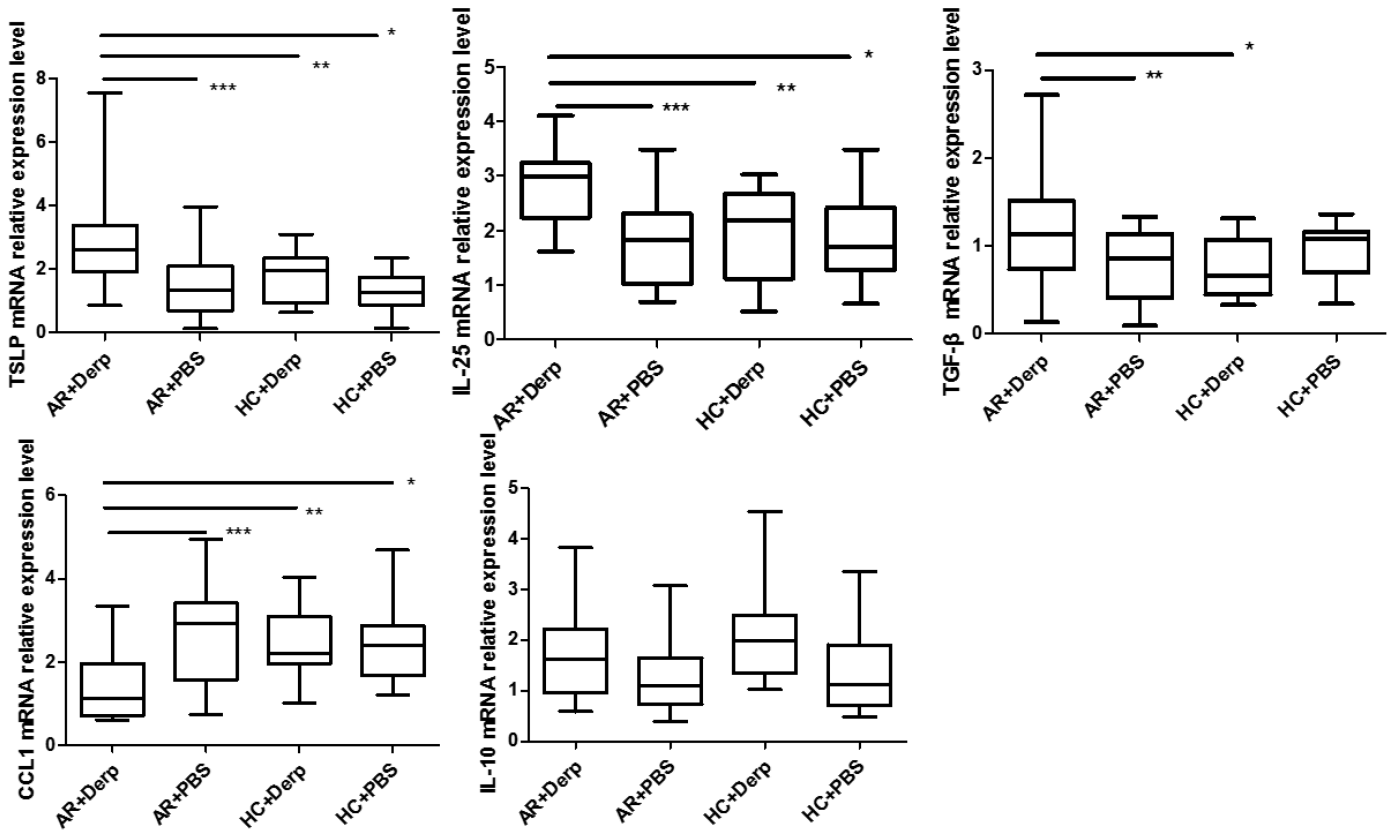
The TSLP concentration in the AR + Derp1 group was higher than that in the other three groups. The CCL1 protein was decreased. There were no significant differences in IL-25, TGF-β, and IL-10. *P <0.05 was considered statistically significant.

**Figure 9:**
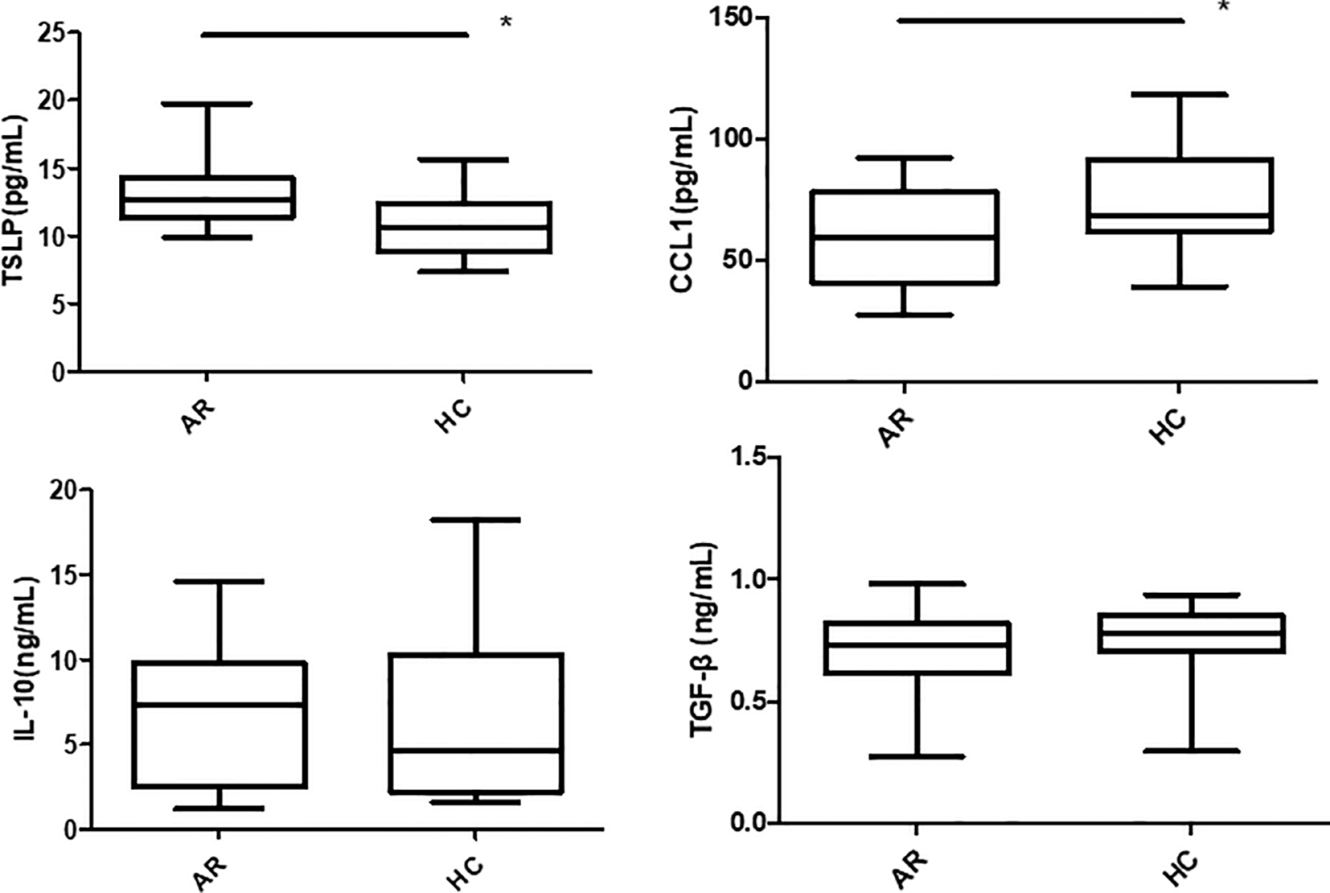
TSLP concentration in the AR group was higher than in the HC group after co-culture. The CCL1 protein was decreased. There were no significant differences in TGF-β and IL-10. *P <0.05 was considered statistically significant.

TSLP, CCL1, IL-25, IL-10 and TGF-β expressions in the pNECs cell suspension were detected by ELISA. The results showed that TSLP concentration was higher in the AR + Derp1 group than in the other three groups (P = 0.000, P = 0.001, and P = 0.000). CCL1 protein expression was decreased (P = 0.058, P = 0.028, and P = 0.012). There were no significant differences in IL-25 (P = 0.904, P = 0.941, and P = 0.846), TGF-β (P = 0.918, P = 0.559, and P = 0.290), and IL-10 (P = 0.681, P = 0.830, and P = 0.746). The mean ± standard deviation of each group is shown in (**Table 3**) and (**Figure 8**). After adding the Tregs co-culture group, IL-10, TSLP, CCL1 and TGF-β expressions in the cell suspension of AR and HC groups after co-culture was detected by ELISA. The trend of the change in expression was similar to that observed in pNECs. The expression level of TSLP increased (P = 0.016), whereas that of CCL1 decreased, with a statistically significant difference (P = 0.047). No significant difference was found between the two groups in IL-10 and TGF-β expressions (P = 0.101 and P = 0.432) (**Figure 9**).

**Table 3:** TSLP, CCL1, IL-25, IL-10, and TGF-β expressions in pNECs suspension by ELISA (x ± s).

	AR+Derp1	HC+Derp1	AR+PBS	HC+PBS
TSLP (pg/ml)	12.16 ± 1.68 **✩✩※※	9.93 ± 1.28	10.53 ± 0.87	10.36 ± 1.04
CCL1 (pg/ml)	65.02 ± 21.30 **※※	84.02 ± 29.54	79.45 ± 22.50	81.73 ± 27.39
IL-10 (ng/ml)	6.24 ± 3.54	6.60 ± 3.08	5.61 ± 5.11	5.70 ± 5.18
TGF-β (ng/ml)	0.17 ± 0.04	0.18 ± 0.05	0.17 ± 0.05	0.15 ± 0.03
IL-25 (ng/ml)	0.78 ± 0.49	0.78 ± 0.03	0.78 ± 0.48	0.77 ± 0.42

AR + Derp1 and HC + Derp1: **, P <0.05. AR + Derp1 and AR + PBS: **✩✩**, P >0.05. AR + Derp1 and HC + PBS: ※※, P <0.01

### Correlation analysis of the flow cytometry and ELISA results

**Figure 10:**
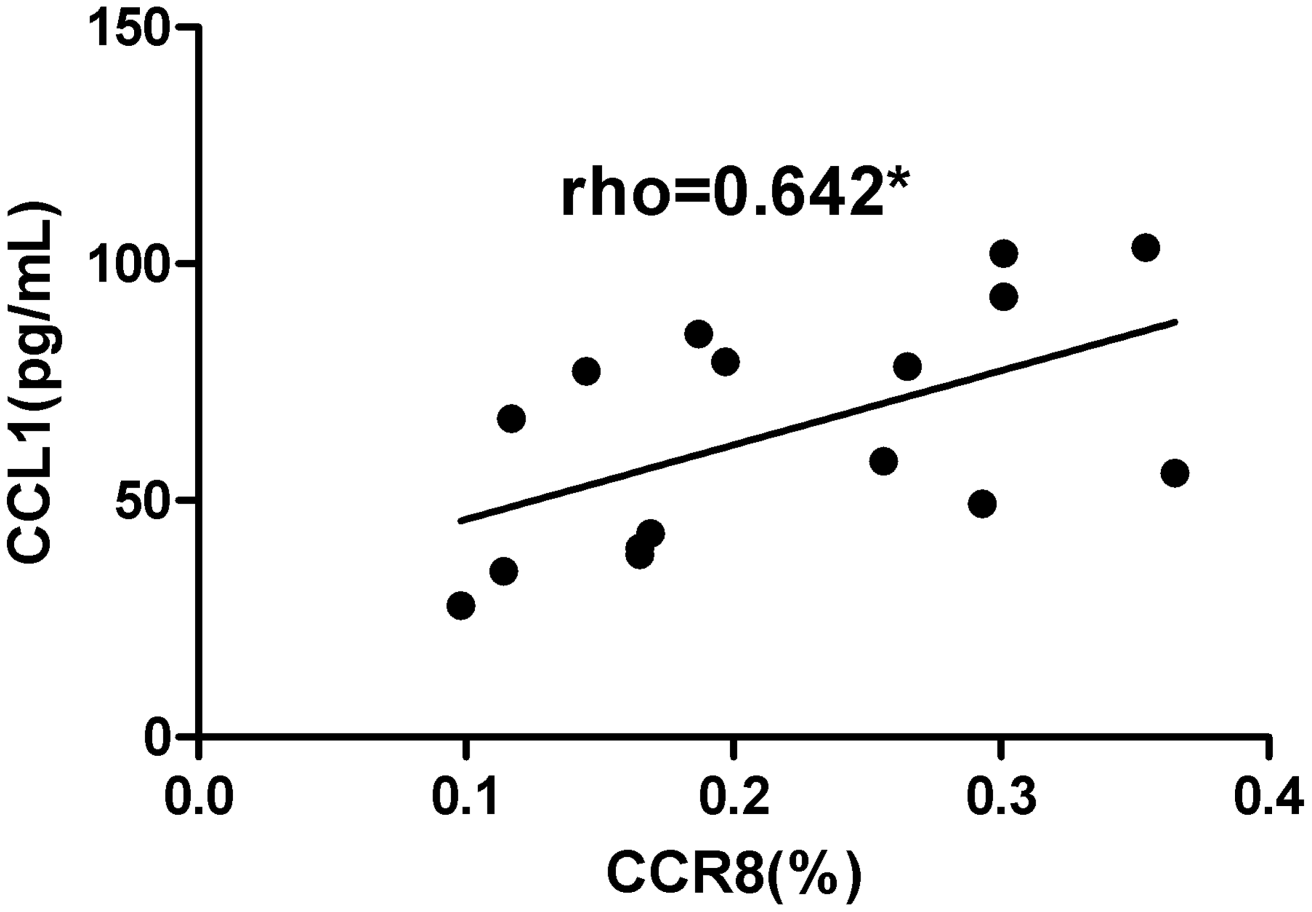
CCL1/CCR8 was positively correlated. *P <0.05 was considered statistically significant.

Correlation analysis was performed between cytokines derived from NECs and surface markers of CD4+CD8+Foxp3+ Tregs in the AR group after co-culture (n = 16), including TSLP/CTLA4, CCL1/CTLA4, TSLP/CTLA4, TSLP/CCR8, and CCL1/CCR8. After co-culture, CCL1/CCR8 levels were positively correlated (r = 0.642, P = 0.007) (**Figure 10**). There were no statistically significant correlations between TSLP/CTLA4 (r = 0.024, P = 0.931), CCL1/CTLA4 (r = 0.344, P = 0.192), and TSLP/CCR8 (r = 0.194, P = 0.471).

## Discussion

AIT is an effective AR treatment that induces clinical tolerance to sensitising allergens, which has been reported for 113 years since 1911 (12). It is administered subcutaneously or sublingually. The immune tolerance mechanisms of AIT are highly complex and far from clear. The induction of Tregs is still considered a pivotal point that can suppress effector T cell responses by early- and late-phase systemic responses (13). However, local tolerance and stability of Tregs in the nasal mucosa remain unexplored. As the first line of the environment, NECs play a vital role in the local immune microenvironment. However, their crosstalk with Tregs is unknown. Understanding the relationship between local Tregs and NECs may improve their therapeutic use for AR.

The nasal mucosa is a local tissue that lacks lymph nodes. The distribution of Foxp3+ Tregs in the nasal mucosa was observed using Foxp3 immunohistochemical staining. Our results revealed that these cells were distributed in the inferior turbinate mucosa of patients with AR and HCs. However, there was no significant difference between the two groups. A previous study showed that patients with AR present low Tregs in the peripheral blood and the nasal mucosa (14). Shirasaki et al. (15) and Sogut et al. (16) suggested that Foxp3+ Tregs play an essential role in the immune microenvironment of the nasal mucosa in AR and can locally regulate the Th1/Th2 balance in the mucosa. Muller et al. (17) also performed immunohistochemical staining on the nasal mucosal tissues of patients with pollen allergy, pollen and dust mite multiple allergies, and HCs and obtained similar results. We conducted a mixed culture of primary NECs and CD4+CD25+Foxp3+ Treg cells *in vitro*. A previous review showed many limitations to animal experiments, especially the small number of epithelial and Treg cells. Therefore, we did not conduct animal experiments.

NECs-derived cytokines modulate innate and acquired immune responses consisting of TSLP and IL-25. Additionally, Treg-associated factors include IL-10 and TGF-β (18, 19). Foxp3+ Tregs infiltrate and expand via chemokine- and cytokine-dependent mechanisms. CCL1 in brain tissue is crucial for Treg accumulation by CCR8 on Tregs (20). TLSP also influences Treg function in tumours (21). This study investigated the potential association between NECs-derived cytokines and Tregs, including TSLP, IL-25, IL-10, TGF-β, and CCL1. Foxp3 and CTLA-4 are vital components of Tregs and are responsible for maintaining immune homeostasis (22, 23). Following co-culture with normal NECs, although there was no difference in Foxp3, which marked the differentiation of Tregs, CCR8 marked the increase in CTLA-4 migration and expression. This indicates that the inhibitory activity was substantially increased. These results suggest that information transmission occurs between NECs and Foxp3+ Tregs by inducing Treg chemotaxis and enhancing their inhibitory activity. Colantonio et al. (24) investigated local Foxp3+ Treg cells in the skin and concluded that the skin exerts its inhibitory activity through chemotaxis toward Tregs via the CCL1/CCR8 pathway. Another study on bile duct sclerosis (25) suggested that IgG-associated CCL1 chemotaxis of Th2 and Foxp3+ Treg cells occurs via the CCL1/CCR8 pathway, although these studies did not focus on airways and allergies.

Furthermore, we investigated the potential association of NEC-derived cytokines with Tregs in NECs of specific house dust mite AR following Derp1 stimulation. CCL1 was detected in NECs and extracellular cells. CCL1 mRNA expression and protein levels in AR cell suspensions decreased after exposure to specific allergens. Although no further evidence supports the involvement of CCL1 derived from the airway mucosal epithelium in allergic reactions in AR pathogenesis, our results indirectly suggest that CCL1 derived from NECs has a regulatory effect on Tregs. IL-10 and TGF-β have a bidirectional regulation effect that regulates the immunosuppressive activity of Foxp3+ Treg. Moreover, although there were no crucial results, they are the main functional cytokines of immunosuppressive activity secreted by Foxp3+ Tregs (26, 27). TSLP suppressed the functional activity of Tregs in the respiratory tract of an animal model (28, 29). Although IL-10, TGF-β, and TSLP are all important cytokines of NECs, our results showed that TSLP expression in NECs increased in the AR group. There was no significant correlation between TSLP, CCR8, and CTLA-4 expressions. Owing to the small number of cells and fixed number of flow cytometry staining channels, we could not detect the expression of the TSLP receptor (TSLPR) on the surface of CD4+CD25+Foxp3+ Tregs. NECs cannot avoid transmitting information via CD4+CD25+Foxp3+ Tregs through TSLP/TSLPR, similar to the lower airway in asthma (30).

Our co-culture results with or without AR stimulated by Derp1 showed that CCL1 derived from NECs was significantly and linearly correlated with CCR8 expression on the surface of CD4+CD25+Foxp3+ Tregs in patients with AR. CCR8 is specifically expressed in Th2 and Tregs and plays three immunological roles: binding to ligands with high affinity, chemotaxis of Tregs to local tissues, and migration to antigen-presenting cells (31–33). Based on our results, the CCL1/CCR8 pathway is one of the information transmission pathways between NECs and CD4+CD25+Foxp3+ Tregs involved in AR pathogenesis. After NECs were exposed to specific allergens, the CCL1 expression level decreased, similar to the CCR8 effect on Tregs. It means that, NECs could enhance the inhibitory function of Tregs via chemotaxis in Th2 immune responses, including but not limited to AR. Local Foxp3+ Tregs in the skin (34) have been investigated, and the results indicated that skin epidermal cells could exert inhibitory activity through chemotaxis to Tregs via the CCL1/CCR8 pathway. Another study on bile duct sclerosis (25) suggested that IgG-associated CCL1 chemotaxis involves Th2 and Foxp3+ Tregs via the CCL1/CCR8 pathway.

**Figure 11:**
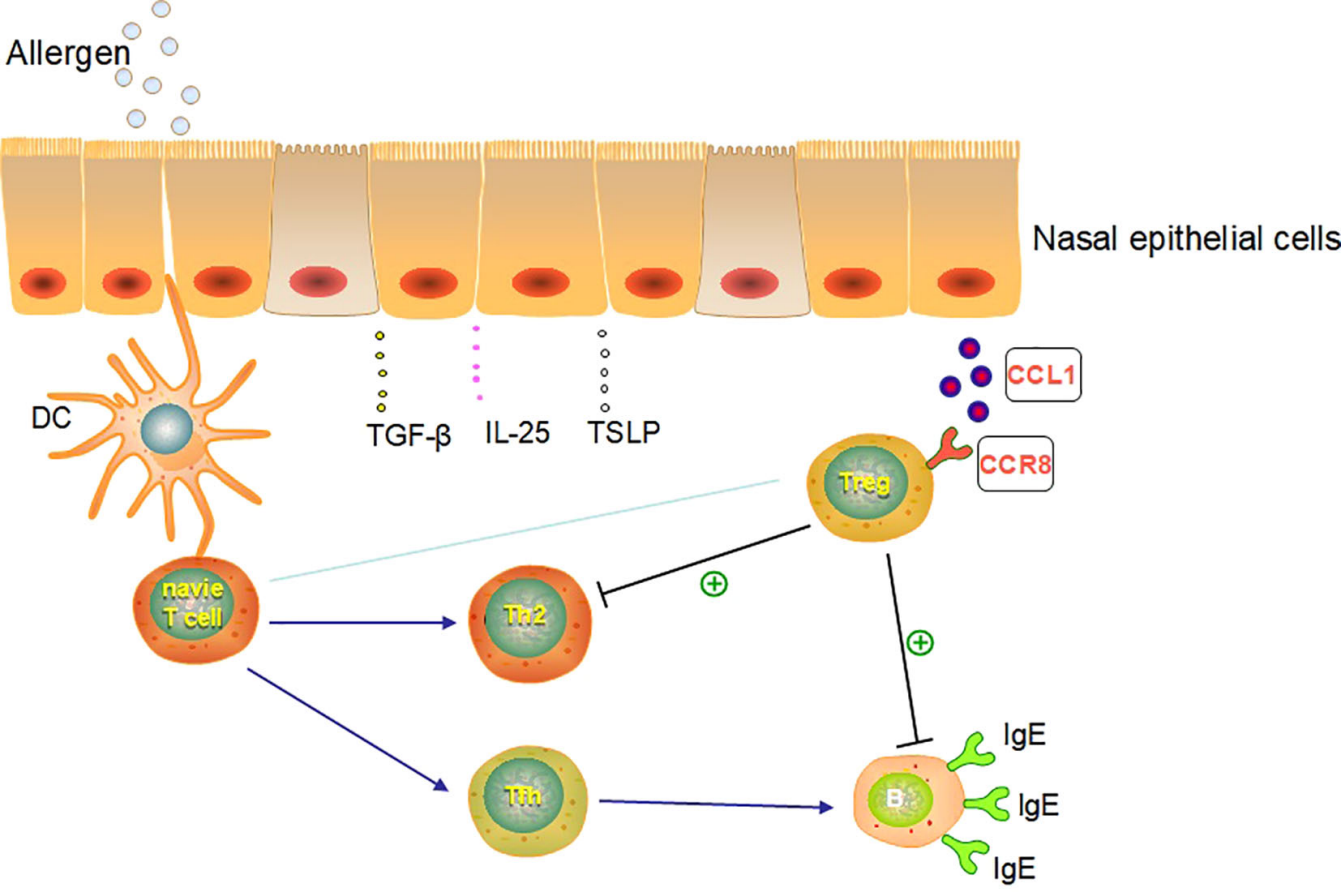
The putative pathway and molecular networks linked with NEC and Treg in AR.

Our results suggest that human pNECs could communicate with Tregs directly and that the suppressive function of Tregs was maintained or mounted after co-culture with NECs. This may become a new target for biological therapy in AR. CCL1/CCR8 may be the pathway between NECs and Tregs *in vitro* and may play a key role in the immune network of AR. These conclusions help identify novel therapeutic targets for AR and airway allergic diseases (**Figure 11**).

## Data Availability

The original contributions presented in the study are included in the article/**Supplementary Material**. Further inquiries can be directed to the corresponding author.
